# The influence of the Japanese waving cat on the joint spatial compatibility effect: A replication and extension of Dolk, Hommel, Prinz, and Liepelt (2013)

**DOI:** 10.1371/journal.pone.0184844

**Published:** 2017-09-14

**Authors:** Lydia Puffe, Kerstin Dittrich, Karl Christoph Klauer

**Affiliations:** Department of Psychology, Albert-Ludwigs-University Freiburg, Freiburg, Germany; Ludwig-Maximilians-Universität München, GERMANY

## Abstract

In a joint go/no-go Simon task, each of two participants is to respond to one of two non-spatial stimulus features by means of a spatially lateralized response. Stimulus position varies horizontally and responses are faster and more accurate when response side and stimulus position match (compatible trial) than when they mismatch (incompatible trial), defining the social Simon effect or *joint spatial compatibility effect*. This effect was originally explained in terms of action/task co-representation, assuming that the co-actor’s action is automatically co-represented. Recent research by Dolk, Hommel, Prinz, and Liepelt (2013) challenged this account by demonstrating joint spatial compatibility effects in a task-setting in which non-social objects like a Japanese waving cat were present, but no real co-actor. They postulated that every *sufficiently salient* object induces joint spatial compatibility effects. However, what makes an object *sufficiently salient* is so far not well defined. To scrutinize this open question, the current study manipulated auditory and/or visual attention-attracting cues of a Japanese waving cat within an auditory (Experiment 1) and a visual joint go/no-go Simon task (Experiment 2). Results revealed that joint spatial compatibility effects only occurred in an auditory Simon task when the cat provided auditory cues while no joint spatial compatibility effects were found in a visual Simon task. This demonstrates that it is not the *sufficiently salient* object alone that leads to joint spatial compatibility effects but instead, a complex interaction between features of the object and the stimulus material of the joint go/no-go Simon task.

## Introduction

In daily life, we are constantly requested to perform a variety of tasks in order to reach specific goals and meet our needs. In many cases, tasks need to be shared or coordinated with other persons, for example while playing a tennis match, performing standard dancing, or carrying heavy furniture. It is therefore not surprising that there has been a growing interest in the last years to investigate cognitive processes that accompany these joint action situations (for an overview see [1–3]).

A prominent paradigm for addressing this issue is the joint go/no-go Simon task, developed by Sebanz, Knoblich, and Prinz [4]. This task is an extension of the classical Simon task [5] (see also [6]), wherein a participant responds in a forced two-choice design to a discrete stimulus feature (e.g., colour, shape, tone pitch) by pressing two spatially arranged response keys (e.g., green stimulus—left key, red stimulus—right key). In addition to this task-relevant stimulus feature, the stimulus position varies randomly, which reflects a task-irrelevant stimulus feature. As a consequence, compatible and incompatible trials occur defined by a match and mismatch, respectively, of response key and stimulus position (in the above example, a compatible trial is given when a green/red stimulus appears on the left/right side of the computer screen; an incompatible trial when a green/red stimulus appears on the right/left side). It turns out that task performance is influenced by the spatial correspondence between the task-irrelevant stimulus position and the required response side (key alignment), resulting in faster responses and fewer errors in cases of compatible trials, and longer reaction times and more errors in incompatible trials, defining the so-called *Simon effect*, also known as *spatial compatibility effect* (SCE; [7], see for an overview [6]).

One of the most accepted and comprehensive explanations for this SCE is given by Kornblum, Hasbrouq, and Osman [8]. According to their *dimensional overlap model*, an overlap between the task-irrelevant spatial stimulus dimension and the spatial orientation of the response keys leads to an interference at the response selection stage. The task-irrelevant spatial stimulus dimension automatically activates a response that either corresponds to or interferes with the correct response as required by the instruction (for alternative accounts, see also [9–12].

Interestingly, when a participant reacts to only one bit of the stimulus information (e.g., to one colour) with one single key (*individual go/no-go task*), the SCE disappears (see for example [13]). The response key is no longer spatially represented due to the absence of another horizontally aligned, task-relevant response key; instead of spatially representing the responses, mental representations in terms of non-spatial “react” or “withhold” codes are conceivable [9]. Thus, the response dimension does not interfere with the irrelevant spatial stimulus dimension anymore [13]. Importantly, when this go/no-go task is performed alongside another participant (*joint go/no-go task*), the SCE reappears—although participants each still react to only one bit of the stimulus information with one single response key; the reappearance of the effect is called *social Simon effect* or *joint spatial compatibility effect* (joint SCE; [4]).

By now, several approaches were developed to explain this reappearing SCE in a joint setting. The *action/task co-representation account* assumes that individuals also co-represent their partner’s task or action plan in addition to their own [4, 14–16]. The content of this representation is whether and when a co-actor is supposed to respond [16]. Specifically, Sebanz and colleagues [4] assume that observed actions and self-performed actions are represented in a functionally equivalent way (see also [17]). Thus, representing one’s own and the partner’s stimulus-response rules results in a fully represented response set. In consequence, this leads to response selection difficulties in cases of incompatible trials, comparable to response selection difficulties in the classical forced two-choice task [4] (but see also [18]). Following this line of argumentation, the action/task co-representation approach explains a reappearing joint SCE exclusively by the social context of the task setting.

As an alternative to the action/task co-representation approach, spatial, non-social explanations have been developed [19 –24]. For example, in the *spatial response coding account*, Dittrich and colleagues [20] assumed that participants code their reaction in accordance to a spatial reference point (e.g., left of…, right of…), as long as this spatial reference point (e.g., the co-actor in a joint go/no-go task setting) is seen as being a part of the task. This spatial coding results in an overlap of the spatial response dimension (e.g., key alignment) and the task-irrelevant spatial stimulus dimension (e.g., stimulus position in the Simon task), leading to interference in cases of incompatible trials. Another more general approach that also stresses the relevance of spatial information for explaining joint SCEs is discussed by Dolk and colleagues [22, 23]. The authors proposed a *referential coding account* that is based on the Theory of Event Coding [11, 25]: Perceiving an event or object will automatically lead to an encoding of specific cognitive feature codes (red, left, small, fast, silent, etc.), that are stored in so-called event files. Thus, the presence of a salient co-actor or any attention-attracting object in a joint go/no-go Simon task causes an automatic representation of an alternative code network. This might include for example a certain stimulus feature or response key, which is assigned to the co-actor or attributed to the object. Thus, network representations of the own and the co-actor’s network comprise highly similar codes. This requires the participant to discriminate accurately between “self” codes (leading to one’s own action) and “other” codes in order to react appropriately. In this situation, the spatial dimension (e.g., left and right) reflects a suitable discrimination criterion, given that it is the most obvious and often the only shared dimension [26, 27]. Giving more weight to the spatial response dimension then causes interference with the irrelevant spatial stimulus position in case of incompatible trials [28]. In individual go/no-go settings, on the other hand, no relational coding occurs because no conflicting alternative codes are available; in line with the finding that SCEs are typically not observed in this condition.

In contrast to the action/task co-representation account, both spatial accounts assume that the social component (the co-actor) modulates the joint SCE by enhancing the spatial interpretation of the situation [20]. This raises the question whether a socially interacting object is at all necessary for producing a joint SCE. Dolk and colleagues [22] addressed the issue by requesting participants to perform an auditory “joint” go/no-go Simon task in the presence of a non-social object. The objects, decreasing in their “extent of socialness”, were a Japanese waving cat, a rotating clock, and either a clicking or a silent metronome. Performing the task alongside a Japanese waving cat, a rotating clock, or a clicking metronome in fact resulted in a significant joint SCE; a silent metronome did not induce the effect. The terminology “joint” in a go/no-go Simon task involving a non-social, not actively responding object is rather imprecise. Nevertheless, we will use this terminology to differentiate between both go/no-go conditions (individual vs. joint). Thus, the “joint” condition also encompasses task settings where a non-social object instead of a real co-actor is present.

The authors concluded that there is no need to have a human co-actor present for a joint SCE to occur, as long as a *salient* reference object is present that introduces a discrimination problem: “In fact, any representation can create conflict with a representation of the currently (most) relevant response if it is sufficiently active. This implies that the represented event would need to be attended and/or sufficiently salient, which obviously applies to a Japanese waving cat or a ticking metronome […]” ([22], p.7). The authors demonstrated striking evidence for a reliable joint SCE in a joint go/no-go Simon task with several non-social objects, but aside from this finding, their study raises another interesting issue: When is (or which features make) an object *sufficiently salient*?

A study that provided first insights into this research question was reported by Lien, Pedersen, and Proctor [29]. Given salient non-social objects are appropriate to function as a source of referencing, is it then possible that a task-irrelevant object is *salient enough* to provide an additional spatial code in a forced two-choice task? The authors assumed a salient, though task-irrelevant object to induce implicitly a second reference frame that should lead to a more pronounced spatial compatibility effect in a forced two-choice task compared to the effect found in a joint go/no-go task. Therefore, they used a procedure similar to the procedure reported by Dolk et al. (2013), Experiment 1. Participants performed an auditory Simon task that comprised a joint go/no-go part and a forced two-choice part (with task type order counterbalanced), while a Japanese waving cat was either present or absent during each task type, resulting in four within-subject conditions. The order of the cat’s presence within each task type was randomized. Participants were seated centrally in front of the computer screen either operating the left and the right response key (forced two-choice task) or operating only the right key (joint go/no-go task). The cat was placed in a distance of 50 cm from the right response key in the cat-present condition, while the position remained empty in the cat-absent condition. The authors replicated the joint SCE in the auditory joint go/no-go Simon task when a Japanese waving cat was present. Additionally, the cat’s presence did not substantially influence the occurrence of a SCE in an auditory forced two-choice task. Most important for our work was their second experiment, in which they asked participants to perform a joint go/no-go task and a forced two-choice task with visual stimuli. Results regarding the forced two-choice task revealed no significant influence of the cat’s presence on the SCE again. But in contrast to their first experiment, the presence of the Japanese waving cat also had no influence on the performance in the visual joint go/no-go Simon task; no joint SCE occurred in the cat-present condition (note that [30] found an effect in a visual joint go/no-go Simon task with a virtual waving cat; for a discussion of their results see General discussion). This might indicate that it is not the *sufficiently salient* object alone that leads to SCEs but instead, a complex interaction between features of the object, stimulus material as well as task type. Perhaps, the potential of an object to produce SCEs in a joint go/no-go Simon task, i.e., its salience, is driven by a correspondence of attention-attracting features of the object (e.g., *visual*, *auditory*, or *social* cues) and certain task properties (e.g., *modality*: visual or auditory Simon task version; *type*: forced two-choice task, individual or joint go/no-go setting).

Looking more closely at the original experiment by Dolk and colleagues [22] revealed that the Japanese waving cat and a rotating clock both provided visual and auditory cues (*cat*: “Participants were able to see the cat […] and to hear the (unpredictable and nonmetrical) sound produced by the waving.”, p.3; *clock*: “[…] a golden clock that contained a visible continuously rotating element and emitted an audible ticking sound […]”, p.5), while the metronome provided just auditory cues (“[…] produced some sort of clicking sound, but did not move in any way.”, p.6). All objects led to a reliable SCE, but it remains unclear whether a specific cue (e.g., auditory, visual) or a combination of cues has been specifically responsible for inducing the joint SCE. The Japanese waving cat for example provided the most pronounced joint SCE (= 19 ms) and revealed in fact several potentially relevant cues (see below). However, Dolk and colleagues [22] varied the non-social objects across experiments. In consequence, it is difficult to compare the cues’ influences as well as the resulting objects’ salience.

Dolk and colleagues [22] discussed that social features (such as the cat’s face, movement of the paw) might have promoted the cat’s salience by implying a certain degree of socialness, priming the concept of a human co-actor that results in an enhanced event code discrimination difficulty (see [22], discussion of Experiment 1, p.5; General discussion, p.11). On the other hand, in line with the above argumentation it might also be possible that the presentation of auditory and visual attention-attracting cues made the object salient because it (partially) matched the task modality. Specifically, the cat produced attention-attracting auditory sounds that might have interfered with the auditory stimulus material. This might have led participants to actively ignore or inhibit the source of distraction (i.e., the cat) which in turn resulted in a spatial representation of the cat.

In order to shed light onto the question of what defines salience of a reference object, we scrutinized which specific cues of the Japanese waving cat influence its salience; that is which cues lead to SCEs in a joint go/no-go Simon task. More specifically, our research question is whether a correspondence of certain cues by the cat and the Simon task modality contributes to the occurrence of reliable joint SCEs. Therefore, the experimental setting and procedure followed the procedure described by Dolk and colleagues [22] as closely as possible; deviations from the original procedure will be indicated. We implemented a joint go/no-go Simon task either with auditory (Experiment 1) or visual material (Experiment 2), wherein a Japanese waving cat was used as a non-social object. Four conditions were implemented: The cat presented either both visual and auditory cues (replication of Dolk and colleagues [22]), an auditory cue, a visual cue, or neither a visual nor an auditory cue. We expect a SCE when cues correspond to the task modality and a reduced or no effect in the other two conditions, when a non-corresponding cue or no cues are provided.

## Experiment 1

Participants performed an auditory joint go/no-go Simon task in the presence of a Japanese waving cat, positioned on the left-hand side of the participant. Four between-subject conditions were realized: Either, the cat both waved and made a sound (VA, visual and auditory cues), made a sound without waving (A, auditory cue), solely waved without a sound (V, visual cue), or neither made a sound nor waved (C, control condition). We expected to find a significant SCE in the VA condition, when two attention-attracting cues of the non-social object are presented that (partly) match the given stimuli during the joint go/no-go Simon task. Note that this would replicate the results of Dolk and colleagues [22]. A reliable SCE should also be observable for condition A, when just an auditory cue is presented. A smaller effect might occur in condition V, when just a visual attention-attracting cue is available. Larger effects for condition A in comparison to condition V would indicate that the correspondence of the object’s cues with the task modality (partially) defines the object’s salience. No effect is expected for the control condition (C). Note that a missing effect in the control condition would demonstrate that only static social cues of the cat (for example the cat’s face) are not sufficient to produce joint SCEs.

### Method

#### Ethics statement

Both studies were carried out in strict accordance with the ethical principles as formulated in the WMA Declaration of Helsinki. If research objectives do not involve issues regulated by law (e.g., the German Medicine Act [Arzneimittelgesetz, AMG], the Medical Devices Act [Medizinproduktegesetz, MGP], the Stem Cell Research Act [Stammzellenforschungsgesetz, StFG] or the Medical Association’s Professional Code of Conduct [Berufsordnung der Ärzte]), then no ethics approval is required for social science research in Germany. Both studies had no such objectives, and therefore, no IRB approval or waiver of permission was sought for these studies.

Participation in both studies was entirely voluntary. All participants gave their written informed consent beforehand to participate in the study. They were informed of the study procedures, of their anonymity, and they were told that they were free to leave the study at any point if they wished to. They were naive regarding the purpose of the experiment and were debriefed about the experimental hypotheses at the end of the experiment. These procedures were in accordance with the German Society for Psychology’s research standards (Grundsätze der Forschung am Menschen, C.III, para. 6).

#### Participants

A group of 106 undergraduate University of Freiburg students participated in the experiment for course credit or as paid volunteers (75 females; 18–39 years, mean age = 22.95 years, *SD* = 4.14). All participants had normal or corrected-to-normal vision, and reported the absence of neurological or hearing problems. Participants needed to satisfy the right-handedness criterion assessed by the Edinburgh Inventory scale [31]. They were classified as”right-handed” by scoring between +58 and +100 points (at least 7 out of 12 items answered with “done with right hand only”). One participant did not meet the handedness criterion and was excluded from the analyses. Fifteen participants were excluded from further analysis based on Tukey’s outlier criterion: Their error rates ranged between 13.1% and 98.5%. One reason for this high dropout might be that participants did not understand the instruction appropriately. No error feedback was given following to the original procedures by Dolk and colleagues [22], in consequence these participants performed the wrong task during the whole experiment (reacted to the no-go stimulus). The final sample consisted of 90 participants (64 female; 18–39 years, mean age = 22.80 years, *SD* = 4.02), distributed per condition as follows: 21 participants in visual and auditory condition (VA), 23 participants in auditory condition (A), 22 participants in visual condition (V), 24 participants in control condition (C). Participants were randomly assigned to one out of four between-subject conditions.

#### Materials and apparatus

**Fig 1:**
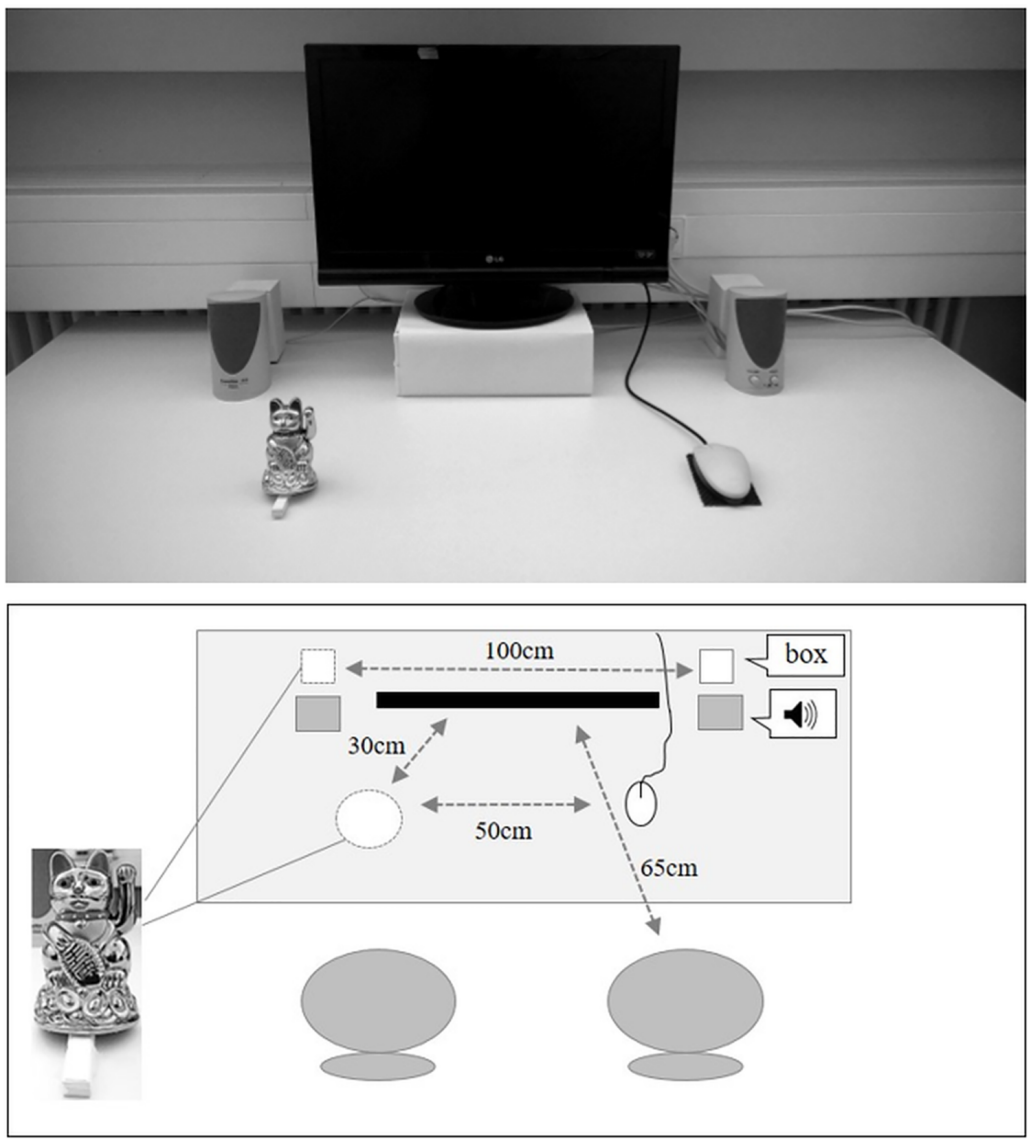
The experimental setup. Participants performed the joint go/no-go Simon task (Experiment 1 –auditory, Experiment 2 –visual) next to a Japanese waving cat that either produced sounds and waved (VA), only produced sounds (A), only waved (V), or that emitted neither sound nor waving (C). Note that the condition A used a second waving cat hidden under a white box, placed behind the left loudspeaker. A second empty white box was accordingly placed behind the right loudspeaker to have a symmetrical set-up.

Two auditory signals designed by [32] consisting of the Dutch words “groen” (green, Sound A) and “paars” (purple, Sound B) were used. Both were compressed and played in reversed order, evoking the signals “oerg” and “chap” without any semantically meaningful content. The mapping of the stimuli as either go or no-go stimulus was counterbalanced across participants. Stimuli were presented with a volume of 60dB from either a left or a right loudspeaker that were positioned on the left and right side of the computer screen. Participants were always seated on the right chair. They responded with the index finger of the right hand pressing the interior key of a computer mouse (see [33]). The left hand rested under the table on the participants’ left thigh. The left chair remained empty (see Fig 1 for details).

A Japanese waving cat (height: 12.5 cm, width: 9 cm, depth: 7 cm, apparently, the same model as used by [22]) was placed on the left-hand side of the participant. The cat was visible in the peripheral visual field and waved with a frequency of 0.4 Hz and an angle of 50° in the vertical plane. The automatic, battery driven arm movement produced an unsystematic waving sound (see also [22], p. 3). To implement the four conditions with different cues, we deviated slightly from the set-up reported by [22]: In the VA condition, the cat stood on a small platform (height: 0.9 cm, width: 0.8 cm, length: 5.2 cm), that caused an inclined position neither affecting the waving movement nor the waving sound. For the V condition, the incline of the cat was strengthened, causing the unsystematic waving sound to disappear, while the cat was visibly waving. Further, for condition A, a second Japanese waving cat was hidden inside a white box, placed behind the left loudspeaker (see Fig 1). Therefore, a rather unsystematic (battery caused) knocking sound was audible when the cat’s path tapped against the wall of the box. The visible cat remained immobile without visible waving movements. Importantly, in all conditions, the same Japanese waving cat was used; the visible cat was present the whole time. Further, diverging from Dolk and colleagues [22], no investigator was present during the session. Regarding the instructions, special attention was given to avoid any spatially related words, like “left” or “right” to prevent a biased response coding (see also [20, 34]).

#### Procedures

The experiment started with a short training phase consisting of eight trials (4 go trials and 4 no-go trials; including 2 compatible and 2 incompatible trials for each trial type in randomized order). The experimental phase consisted of four experimental blocks of 128 trials each, comprising 64 go trials and 64 no-go trials (including 32 compatible go trials—the go stimuli sounded from the right loudspeaker, and 32 incompatible go trials—the go stimuli sounded from the left loudspeaker). Each trial started with a warning sound presented for 300 ms, the go or no-go stimulus followed after 700 ms and was presented for 300 ms. The next trial followed after the response was given or after 1700 ms had passed, whichever occurred first; the inter-stimulus-interval was 1000 ms. Participants were instructed to focus on a white fixation cross that remained visible in the centre of the computer screen throughout the experiment. Participants were to react as fast and accurate as possible to the assigned go stimulus, irrespective of whether it sounded from the left or right loudspeaker. The entire experimental session took approximately 40 minutes.

### Results

Error trials (trials with erroneous go or no-go responses) as well as reaction times below 150 ms and outliers were omitted from each individual reaction time distributions. Outliers were identified by Tukey’s criterion (i.e., reaction times 1.5 times the interquartile range below the first quartile or reaction times above the third quartile; [35]). This resulted in an exclusion of 0.81% of all trials.

Trials were coded as compatible or incompatible. Accuracy data (percentage of trials without erroneous go or no-go responses) were arcsine-transformed for the statistical analyses. For easier interpretation, untransformed error rates will be reported in the tables. Mean reaction times and mean error rates for compatible and incompatible trials are presented separately for conditions (VA, A, V, and C), see Table 1.

**Table 1:** Results of Experiment 1. Mean reaction times (ms), error rates (%), and standard deviations for compatible and incompatible trials as a function of condition in the auditory joint go/no-go Simon task.

Condition	Spatial compatibility								
	Compatible		Incompatible				Joint SCE		
	*M*	*SD*	*M*		*SD*		*M*		*SD*
	**Reaction times**								
Waving and sound (VA)	584	79	594	76		10*		18	
Sound (A)	573	69	579	68		5*		10	
Waving (V)	595	88	599	90		3		12	
No sound, no waving (C)	590	64	587	67		-3		17	
**Overall**	586	74	589	75		4*		15	
	**Error rates**								
Waving and sound (VA)	2.1	2.6	1.4	1.4		-0.7		1.6	
Sound (A)	2.0	1.7	1.7	1.8		-0.3		1.0	
Waving (V)	1.5	1.2	1.4	1.0		-0.1		0.9	
No sound, no waving (C)	2.0	2.3	2.2	2.0		0.2		1.1	
**Overall**	1.9	2.0	1.7	1.6		-0.2		1.2	

SCE = Spatial compatibility effect defined as the reaction time and error difference of incompatible and compatible trials. Small differences between SCEs and the means from which they are computed reflect rounding error.

* *p* < .05.

Reaction times and accuracy data were submitted to a 2 × 4 repeated-measure analysis of variance (ANOVA) with compatibility (compatible, incompatible) as within-subjects factor and condition (VA, A, V, and C) as between-subjects factor. For reaction times, the ANOVA revealed a significant main effect of compatibility, *F* (1, 86) = 6.24, *p* = .014, η_p_² = .068, indicating faster responses in compatible trials (mean reaction time of *M* = 586 ms, *SD* = 74) than in incompatible trials (mean reaction time of *M* = 589 ms, *SD* = 75). Further, an interaction of compatibility and condition was found, *F* (3, 86) = 2.95, *p* = .037, η_p_² = .093. As expected, the SCE differed between conditions (see Table 1). Follow up *t*-tests revealed a significant SCE for condition VA, *t*(20) = 2.54, *p* = .019, *d* = 0.80, as well as for condition A, *t*(22) = 2.48, *p* = .021, *d* = 0.75. No significant SCEs were observed for condition V, *t*(21) = 1.30, *p* = .208, and condition C, *t*(23) = -0.86, *p* = .398. A Tukey post-hoc test did not reveal a significant difference between condition VA (mean SCE = 10 ms, *SD* = 18) and A (mean SCE = 5 ms, *SD* = 10), *p* = .936. The four conditions did not differ in their overall reaction times, *F* < 1. No significant effects were found for the accuracy data (all *F*s < 1.6).

### Discussion

In the first experiment, we scrutinized the influence of attention-attracting cues on the occurrence of reliable auditory joint SCEs. Results revealed a significant joint SCE of 10 ms in the VA condition in which visual and auditory cues were presented simultaneously. This replicates the results of Dolk and colleagues [22]. Further, a reliable joint SCE of 5 ms was found for condition A, when only an auditory cue was presented. Just waving (V) or no cues (C) did not induce a significant joint SCE.

Assuming that the Japanese waving cat is perceived as an extraordinary object in a laboratory setting, it might be argued that the cat itself attracts attention and induces a joint SCE. Accordingly, we should have found reliable joint SCEs in all conditions. Further, it has been speculated that the cat’s face provides social cues that might render the cat salient, inducing a joint SCE [22]. Then again, we should have found reliable joint SCEs in all conditions. Contrary to these predictions, we only found reliable effects in two out of four conditions.

The auditory cue thus had an important influence for the inducement of joint SCEs, because they were only observed in the conditions that involved auditory cues. Descriptively, the joint SCE of the VA condition was larger (10 ms) than the joint SCE of the A condition (5 ms), but the difference was not significant. Thus, a combination with visual cues does not significantly amplify a joint SCE, suggesting that the auditory cue in particular might be of importance here.

As the auditory Simon task implies, participants were requested to focus their attention on auditory stimulus material for an appropriate task performance, but they were at the same consistently distracted by the cat’s additional auditory cue. This correspondence of task stimuli and additional attention-attracting cue in terms of modality might have led participants to strategically ignore the source of distraction (i.e., the cat) to avoid an impeded task performance. In turn, this might have resulted in an emphasized representation of the spatial alignment and may have made the cat sufficiently salient. Note that in condition A, it was not the visual cat that produced the sound but the hidden cat that tapped unsystematically against the wall of the box. Here as well, the waving sound might have attracted attention to the left side of the task set, stressing the spatial dimension of the task setting (for a more detailed discussion, see General discussion).

In a second step, we ask whether the effects are limited to the auditory joint go/no-go Simon task or transfer to the visual modality as well. If joint SCEs induced by non-social objects occur due to a correspondence of cues of the non-social object and the material used in the joint go/no-go Simon task, then joint SCEs might be observable for the VA and V condition in a visual version of the Simon task. As described in the Introduction, Lien and colleagues [29] did not find a joint SCE in their visual go/no-go Simon task when a Japanese waving cat was present (Experiment 2). They speculated that the failure to find an effect might be explained by the material used in the visual go/no-go task that only indicated a spatial direction but did not vary itself in spatial location: Stimuli comprised pictures of a hand presented centrally on the computer screen, which either pointed to the left, right, or straight towards the participant; additionally, a red or green dot was presented in the middle of the hand (see also [4]). Lien and colleagues [29] speculated that “allocating attention to the centrally presented visual target may have reduced the chance of spatial reference being generated from the salient, irrelevant object located in the peripheral field”, (p. 929). Presumably, the presence of a non-social object like a Japanese waving cat might also induce joint SCEs in a visual joint go/no-go Simon task, if stimuli positions vary horizontally on the computer screen, increasing the probability that participants direct their attention to the object. In line with this assumption, Stenzel and Liepelt [30] found reliable SCEs in a visual go/no-go Simon task with different virtual non-social objects; in their experiments, non-social objects were presented more closely to the stimuli of the joint go/no-go Simon task. We conducted a second experiment in order to investigate whether (a) joint SCEs are also observable in a visual joint go/no-go Simon task with horizontally varying stimuli and a non-social reference object and (b) whether a correspondence of the attention-attracting cues of the non-social object and stimuli in the joint go/no-go Simon task also influences the occurrence of reliable SCEs in the visual modality.

## Experiment 2

In this experiment, we used a visual go/no-go Simon task with stimulus material adapted from [19, 20, 36]: Red or green coloured circles were presented to the left or right side within a white horizontally arranged rectangular. In line with the results of Experiment 1, a correspondence of visual attention-attracting cues (provided by the cat’s waving) and visual task stimuli is expected to induce a reliable SCE for the V and VA condition, while no effects are expected to occur in the A and C condition. Setting, procedure, and conditions were comparable to Experiment 1 except for the differences indicated below.

### Method

#### Participants

A new sample of 96 participants was tested (57 females; 17–45 years, mean age = 22.46 years, *SD* = 5.09), all reported an absence of colour-blindness. Three participants needed to be excluded according to Tukey’s outlier criterion applied to reaction times and error rates (with mean reaction time of *M* = 460 ms, *SD* = 180 and mean error rates of *M* = 7.5%, *SD* = 5.5, in the total sample’s distribution of reaction time, *M* = 314 ms, *SD* = 47, and error rates, *M* = 1.5%, *SD* = 2.1). One participant removed the battery of the cat during the experiment; this participant was excluded from analyses. The final sample comprised 92 participants (55 females; 17–45 years, mean age = 22.48 years, *SD* = 5.18), distributed per condition as follows: 23 participants in visual and auditory condition (VA), 23 participants in auditory condition (A), 24 participants in visual condition (V) and 22 participants in control condition (C).

#### Materials and apparatus

The only difference between Experiment 1 and 2 was the visual stimulus material of the joint go/no-go Simon task (adapted from [19, 20, 36]): A white rectangle (subtending a solid angle of 8.5° width × 3.3° height in terms of visual angle) was presented centrally on a black background of a computer screen with a refresh rate of 100 Hz. Within this figure two white framed circles (1° in radius and 3.7° in midpoint to midpoint distance) were shown, appearing in the left half or the right half of the rectangle. Target stimuli were red or green filled circles covering one of the unfilled circles in each trial. The colour assigned to the go stimulus was counterbalanced across participants.

#### Procedures

Each trial started with the presentation of the white frame for 1000 ms (equivalent to the 300 ms warning sound and 700 ms delay of Experiment 1), followed by the coloured stimulus (either go or no-go stimulus) for 300 ms. After the response was given or 1700 ms had passed, the next trial followed after an inter-stimulus-interval of 1000 ms. The rectangle disappeared in this trial phase to keep the trial sequence as similar as possible to Experiment 1 (assuming that the reappearance of the stimulus figure leads to an orientation reaction, equivalent to the warning sound used in Experiment 1).

### Results

Data were pre-processed in the same way as reported for Experiment 1, leading to an exclusion of 0.28% of all trials. A repeated measure analysis of variance for reaction times with compatibility (compatible, incompatible) as within-subjects factor and condition (VA, A, V, and C) as between-subjects factor revealed neither a main effect of compatibility, *F* < 1, nor a significant interaction of compatibility and condition, *F* < 1.0. These results indicate that reaction times in compatible and incompatible trials were not significantly different and also similar across conditions (for more details, see Table 2). No significant results were found for accuracy data, as well.

**Table 2:** Results of Experiment 2. Mean reaction times (ms), error rates (%), and standard deviations for compatible, and incompatible trials as a function of condition in the visual joint go/no-go Simon task.

Condition	Spatial compatibility								
	Compatible		Incompatible				Joint SCE		
	*M*	*SD*	*M*		*SD*		*M*		*SD*
	**Reaction times**								
Waving and sound (VA)	304	29	302	29		-3		11	
Sound (A)	317	32	315	37		-2		14	
Waving (V)	306	26	307	28		1		14	
No sound, no waving (C)	311	30	313	31		3		9	
**Overall**	309	29	309	31		0		12	
	**Error rates**								
Waving and sound (VA)	1.6	1.9	2.0	1.9		0.4		1.1	
Sound (A)	1.3	1.8	1.6	1.8		0.3		0.7	
Waving (V)	1.1	1.6	1.3	1.7		0.2		0.8	
No sound, no waving (C)	1.0	0.9	0.9	0.7		-0.1		0.8	
**Overall**	1.3	1.6	1.4	1.6		0.2		0.9	

SCE = Spatial compatibility effect defined as the reaction time and error difference of incompatible and compatible trials. Small differences between SCEs and the means from which they are computed reflect rounding error.

### Discussion

Experiment 2 was conducted to test the assumption that the salience of a non-social object is primarily linked to a correspondence of attention-attracting visual cues provided by the reference object (here Japanese waving cat) and the material of the visual joint go/no-go Simon task. However, the present results revealed no reliable joint SCE, contrary to the results pattern found in Experiment 1. Neither the conditions including visual waving movements, nor conditions involving auditory cues seem to induce a pronounced salience of the non-social object. Presumably, the cat did not adequately attract participants' attention and was rather easy to ignore, i.e., it was not sufficiently salient.

## General discussion

The present study was conducted to investigate the role of non-social objects as a source of spatial referencing in joint go/no-go Simon tasks. Dolk and colleagues [22] pointed out that any non-social object can induce a joint SCE as long as it is *sufficiently salient*. But it was not defined what makes a non-social object *sufficiently salient*. The authors [22] already speculated that “[…] it is reasonable to assume that different types of co-actors or reference objects differ in salience” (p.10). They used some non-social reference objects that also differed in their attention-attracting cues. This led us to expect that it might be a correspondence of attention-attracting cues provided by a non-social object (e.g., waving sound of a Japanese waving cat) and the modality of the joint go/no-go Simon task (e.g., auditory) that makes an object sufficiently salient to induce a joint SCE; perhaps, this object is then (in case of correspondence) seen as a distracter, leading participants to ignore or inhibit the object’s input, which emphasizes the spatial alignment and facilitates spatial referencing. To test this assumption, we partly replicated the original experiment of Dolk and colleagues ([22], Experiment 1), wherein a Japanese waving cat provided both visual and auditory cues. In order to disentangle the role of the cat’s multiple attention-attracting cues, visual and auditory cues were varied systematically. In Experiment 1, joint SCEs were only found for the conditions in which auditory cues were presented. Providing visual cues in addition to auditory cues did not significantly increase the size of the joint SCE. Moreover, presenting only visual cues or no cues provoked no significant joint SCE. We therefore conclude that a correspondence of the auditory cues and the auditory stimulus material of the task caused a reliable joint SCE. However, we did not find analogous results for a visual joint go/no-go Simon task (Experiment 2). Instead, the reference object did not induce joint SCEs in any condition.

As suggested by an anonymous reviewer, a distributional RT analysis, by means of so-called delta plots (see for details [37–40]), investigated the temporal dynamics of the joint SCE for both task modalities. Details are reported in the supplemental material (S1 Appendix). The analyses adduced no significant evidence for a role of response speed for the magnitude of the observed SCE effects. Given the relatively small sizes of the present SCEs per RT bin, it is probably prudent not to draw strong conclusions from these delta plot pattern.

In what follows, we first discuss the results of Experiment 1 before we turn to the results of Experiment 2. Experiment 1 revealed joint SCEs in an auditory go/no-go task in conditions with auditory cues. Similarly, Dolk and colleagues [22] found significant joint SCEs in an auditory go/no-go Simon task for objects that made sounds: Joint SCEs were found when a Japanese waving cat (auditory and visual cues), a rotating clock (auditory and visual cues), or a metronome (auditory cue) were used as a non-social reference object, but no effect was observed with a silent metronome. We assume that auditory cues given by an object were seen as task-impeding because they correspond to the modality of the attended stimuli in the Simon task. In consequence, participants try to ignore the distracting input of the object, resulting in an emphasized representation of the spatial alignment. In turn, participants code their reactions spatially, in relation to the non-social object, in order to disentangle automatically activated code networks for appropriate reactions [11, 21, 22, 23, 25] or as a result of a rather automatic, spatial attentional process [19, 20].

In our argumentation provided so far, we presume that the auditory attention-attracting cues made the Japanese waving cat sufficiently salient to induce joint SCEs. However, only in the VA condition did the cat produce sounds while in the A condition, the visible Japanese waving cat was silent. To produce sounds in the A condition, a second Japanese waving cat was hidden inside a white box, placed behind the left loudspeaker, that tapped against the wall of the box. We do not know if participants realized that the sound was not made by the visible Japanese waving cat itself. If participants correctly located the tapping sound as originating from the white box, then it might be possible that it was not the Japanese waving cat that served as spatial reference, but the “tapping box”. Note that it is highly likely that we would also have found effects if only the “tapping box” had been present, but no Japanese waving cat. Such a condition would be similar to Experiment 3 of [22] where a ticking metronome led to a SCE.

The VA condition of Experiment 1 closely replicated Dolk and colleagues’ procedure ([22], Experiment 1). However, they reported a joint SCE that was nearly twice as large as the joint SCE found in Experiment 1 (19 ms versus 10 ms). One important difference in the procedure might explain this discrepancy: Participants of Dolk and colleagues [22] performed the joint go/no-go Simon task with the Japanese waving cat present and absent. Either the Japanese waving cat already stood left hand-sided on the table at the beginning of the experiment and was removed after the first half of the experiment, or the Japanese waving cat was placed on the table in the second part of the experiment. Specifically, this latter condition (the cat was placed on the table in the second half of the experiment) might have prompted participants’ attention to the cat. Additionally, when participants observe the experimenter placing the cat on the table, participants might speculate that the cat is task-relevant, additionally strengthen its salience. This “block-wise procedure” might explain the discrepancy in the size of the joint SCE.

The results of Experiment 1 led us to conclude that the correspondence of attention-attracting cues and stimulus material of the Simon task explains joint SCEs in a joint go/no-go Simon task with non-social objects. However, this link seems to be restricted to the auditory modality. In fact, we found no reliable joint SCE in Experiment 2 using a visual joint go/no-go Simon task, whether the cat provided visual waving cues or auditory cues. Our results are in line with findings of [29], who also placed a Japanese waving cat beside participants and found significant joint SCEs in an auditory joint go/no-go Simon task but not in a visual joint go/no-go Simon task. They speculated that by presenting visual stimuli centrally on the computer screen, objects located in the peripheral field are not perceived. Although our visual stimuli of the go/no-go Simon task of Experiment 2 varied in horizontal position on the computer screen, participants might still not have perceived the Japanese waving cat, because they focused their attention around the visual Simon stimulus on the computer screen. A similar focusing of attention was not necessary in the auditory go/no-go Simon task (but note that here as well, participants were asked to look at a fixation cross during the experiment). Instead, both loudspeakers were positioned 1m apart, which might have broadened the attentional focus, including the cat in that focus (see also [22, 29]).

Evidence for the suggestion that the focus of attention explains the missing joint SCE in the visual joint go/no-go Simon task when non-social objects are present was provided by Stenzel and Liepelt ([30], Experiment 1): They used a visual joint go/no-go task and presented a virtual Japanese waving cat in the left lower corner of the computer screen. Granted that the attentional focus is concentrated around the visual Simon stimuli, it is very likely that the virtual, paw-waving cat still fell inside the narrowed attentional field of the participants. Stenzel and Liepelt [30] indeed found reliable joint SCEs. However, the experiment also differed in several other aspects from the present Experiment 2. For example, the Japanese waving cat was animated to make it appear as if the cat would react to the presented stimuli. This uncommon occurrence might have attracted participants’ attention, emphasizing spatial alignment and thereby inducing the effect. In consequence, we can only partly confirm the assumption of the relevance of correspondence between cues of an object and Simon task modality for the occurrence of joint SCEs in joint go/no-go Simon tasks; the missing effect regarding visual stimulus material remains an open issue.

Stenzel and Liepelt [30] explored not only joint SCEs using a Japanese waving cat and visual material, they also investigated whether a constantly visual waving cue is easier to ignore than visual cues with irregular, intermittent movements. Therefore, they manipulated the response mode of some non-social objects (Japanese waving cat, scrambled pattern, or a wooden hand) and human co-actors to either show a continuous movement or an irregular movement mimicking turn-taking responses. Interestingly, joint SCEs were increased in a turn-taking response mode, irrespective of whether it was shown by a virtual Japanese waving cat, scrambled pattern, wooden or human hand. They speculated that non-social objects reacting in a turn-taking mode might be perceived more similar to the reaction given by the participant himself/herself. This is supposed to facilitate agency attribution, leading to increased joint SCEs (for more details see also [41]). Hence, with regard to a non-social object’s salience it also seems to be relevant *how* the reaction is given [30]. In relation to the missing effect in waving conditions of Experiment 1 and 2, the continuous “response mode” of the cat might also have contributed to a diminished joint SCE as a consequence of natural habituation processes.

To conclude, it is not a non-social object per se that is or is not sufficiently salient to induce joint SCEs. For the auditory modality, we instead found evidence that a correspondence between cues of a non-social object and modality of the Simon task affects joint SCEs, presumably because the non-social object reveals cues that need to be ignored in the case of correspondence, thus stressing spatial alignment and causing an increased salience. Several other aspects might affect an object’s salience in terms of serving as a spatial reference in a joint go/no-go Simon task. Therefore, more research is needed to explore these complex dependencies.

## Supporting information

S1 File Dataset and script of main analysis and distributional RT analysis of Experiment 1. (ZIP)

S2 File Dataset and script of main analysis and distributional RT analysis of Experiment 2. (ZIP)

S1 Appendix Detailed information of distributional RT analyses for Experiment 1 and 2. (PDF)
