# Supplementary material for: The influence of the Japanese waving cat on the joint spatial compatibility effect: A replication and extension of Dolk, Hommel, Prinz, and Liepelt (2013)
Source: PLoS One. 2017 Sep 14;12(9):e0184844. doi: 10.1371/journal.pone.0184844 (PMC5599021; doi:10.1371/journal.pone.0184844)
Supplement: S1 Appendix — (PDF) [file pone.0184844.s003.pdf]

## S1 Appendix. Detailed information of distributional RT analyses for Experiment 1 and 2.

It has been discussed elsewhere that SCEs differ in their temporal dynamics depending on task modality (for an overview see [37]). Therefore, we conducted a distributional reaction time (RT) analysis to gain insight whether temporal dynamics contribute to the differences in our results pattern. A delta plot was calculated per task modality [38, 39, 40], that reveals the magnitude of SCEs at five RT bins (quintiles). More specifically, it depicts the difference between two RT distributions across RT bins on the y-axis (i.e., the difference of RT distributions of compatible and incompatible trials, thus revealing joint SCE), as a function of the average of both RT distributions across RT bins (on the x-axis). The slope of the delta plot accounts for RT differences between compatible and incompatible trials (i.e. joint SCE) across response time. A positive slope indicates the joint SCE to be larger for slower responses, whereas a negative slope indicates joint SCE to be larger for faster responses. As can be seen in Fig 2 below, mean RTs differ between both experiments with a slow response speed for the auditory joint go/no-go Simon task (Fig 2A, upper panel) compared to fast responses in the visual joint go/no-go Simon task (Fig 2B, lower panel). This difference in reaction times depending on task modality was likewise reported by Lien, Pedersen, and Proctor [29] (averaged reaction times reported for go/ no-go conditions with the cat being present: $M_{\text{auditory}}$ = 468 ms, $SD_{\text{auditory}}$ = 26; $M_{\text{visual}}$ = 371 ms, $SD_{\text{visual}}$ = 22), indicating that processing and/or response reaction is slower for auditory Simon stimulus material compared to visual stimuli.

Descriptively, a flat positive slope occurred for both task modalities. However, there was no evidence for a significant effect of RT bins on the magnitude of the joint SCE in neither auditory joint go/no-go task, $F$ (1.406, 125.136) = .35, $p$ = .627, $\varepsilon$ = .35, nor the visual go/no-go task, $F$ (1.507, 137.159) = .93, $p$ = .373, $\varepsilon$ = .38. These results diverge from the typical pattern discussed in the literature (see [37] for more details). As such, SCEs in auditory joint go/no-go Simon tasks increase with increasing response reactions, whereas SCEs in visual joint go/no-go Simon tasks usually decrease with increasing reaction times. Note however, that our joint SCEs as a function of mean RT only ranged between 2 ms and 5 ms (Experiment 1, see Fig 2A) and -2 ms and 1 ms (Experiment 2; see Fig 2B), and so it is not surprising that there is no effect of RT bins on these relatively small SCEs.

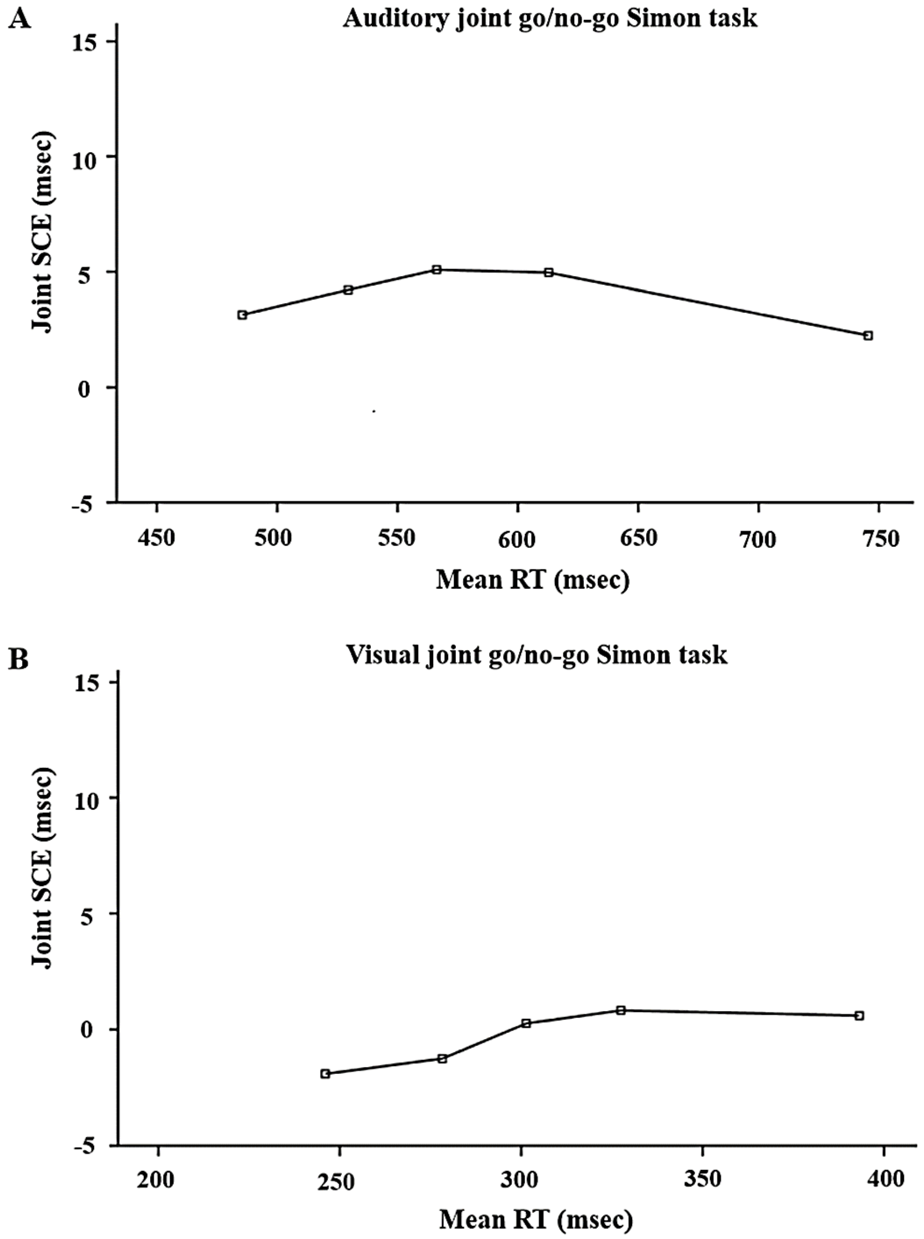


**Fig 2. Delta plot for the auditory and visual joint go/no-go Simon task modality.** Joint SCEs are depicted as a function of mean reaction times per RT bins (quintiles) in A (upper panel) for the auditory joint go/no-go Simon task (Experiment 1) and in B (lower panel) for the visual joint go/no-go Simon task (Experiment 2).
